# Dynamic Functional Connectivity Signifies the Joint Impact of Dance Intervention and Cognitive Reserve

**DOI:** 10.3389/fnagi.2021.724094

**Published:** 2021-09-10

**Authors:** Kristína Mitterová, Martin Lamoš, Radek Mareček, Monika Pupíková, Patrik Šimko, Roman Grmela, Alena Skotáková, Pavlína Vaculíková, Irena Rektorová

**Affiliations:** ^1^Applied Neuroscience Research Group, Central European Institute of Technology, Masaryk University, Brno, Czechia; ^2^Faculty of Medicine, Masaryk University, Brno, Czechia; ^3^Brain and Mind Research Program, Central European Institute of Technology, Masaryk University, Brno, Czechia; ^4^Department of Health Promotion, Faculty of Sports Studies, Masaryk University, Brno, Czechia; ^5^Department of Gymnastics and Combatives, Faculty of Sports Studies, Masaryk University, Brno, Czechia; ^6^First Department of Neurology, Faculty of Medicine, Masaryk University and St. Anne’s University Hospital, Brno, Czechia

**Keywords:** cognitive reserve, dance intervention, dynamic resting-state functional connectivity, attention, bottom-up processing, top-down processing, dwell time, coverage

## Abstract

Research on dance interventions (DIs) in the elderly has shown promising benefits to physical and cognitive outcomes. The effect of DIs on resting-state functional connectivity (rs-FC) varies, which is possibly due to individual variability. In this study, we assessed the moderation effects of residual cognitive reserve (CR) on DI-induced changes in dynamic rs-FC and their association on cognitive outcomes. Dynamic rs-FC (rs-dFC) and cognitive functions were evaluated in non-demented elderly subjects before and after a 6-month DI (*n* = 36) and a control group, referred to as the life-as-usual (LAU) group (*n* = 32). Using linear mixed models and moderation, we examined the interaction effect of DIs and CR on changes in the dwell time and coverage of rs-dFC. Cognitive reserve was calculated as the residual difference between the observed memory performance and the performance predicted by brain state. Partial correlations accounting for CR evaluated the unique association between changes in rs-dFC and cognition in the DI group. In subjects with lower residual CR, we observed DI-induced increases in dwell time [*t*(58) = –2.14, *p* = 0.036] and coverage [*t*(58) = –2.22, *p* = 0.030] of a rs-dFC state, which was implicated in bottom-up information processing. Increased dwell time was also correlated with a DI-induced improvement in Symbol Search (*r* = 0.42, *p* = 0.02). In subjects with higher residual CR, we observed a DI-induced increase in coverage [*t*(58) = 2.11, *p* = 0.039] of another rs-dFC state, which was implicated in top-down information processing. The study showed that DIs have a differential and behaviorally relevant effect on dynamic rs-dFC, but these benefits depend on the current CR level.

## Introduction

Some lifestyle factors are known to ameliorate the risk of cognitive decline and dementia. A study by Fratiglioni et al. (2004) proposed three factors that play this role, namely, social network, cognitive leisure, and physical activity. Dance interventions (DIs) represent a unique synergy of these three factors, which can affect a variety of age-related outcomes, including reducing the risk of falls (Rodrigues-Krause et al., 2016), decreasing depression (Marks, 2016), and influencing other fitness parameters associated with white matter integrity changes (Šejnoha Minsterová et al., 2020). Concerning cognition, research has shown that DIs have compelling benefits for the memory (Rehfeld et al., 2018), attention (Coubard et al., 2011), and psychosocial domains (Ehlers et al., 2017). A recent meta-analysis concluded that DIs deliver the strongest effects on global cognition and memory compared with not exercising or walking, but they do not have the same effect on inhibition or the task-switching aspects of executive function (Meng et al., 2020). In our previous study, we described improved figural fluency resulting from a 6-month DI as compared with the life-as-usual (LAU) condition (Kropáčová et al., 2019). Figural fluency evaluates the ability of executive functions to provide information about divergent reasoning, divided attention, planning, and mental flexibility (Johanidesová et al., 2014).

In terms of functional brain changes, exercise interventions generally led to increases in the resting-state functional connectivity (rs-FC) between the regions of the default mode network (DMN) (Li et al., 2014), between the DMN and motor networks (McGregor et al., 2018), and within the sensorimotor and frontoparietal networks; in the latter finding, exercise intervention was also associated with improved executive function (Voss et al., 2010). In a study by Rosano et al. (2010), physical activity treatment led to higher activations within the dorsolateral prefrontal, posterior parietal, and anterior cingulate (ACC) cortices of the executive control network during a digit symbol substitution test. As for the effects of DIs, the extent of our research showed that there is only one resting-state fMRI study that assessed the amplitude of low-frequency fluctuations (ALFFs) before and after a 3-month-long intervention in a group of 19 subjects with mild cognitive impairment (MCI). This study then found increased ALFFs in the frontotemporal, entorhinal, ACC, and parahippocampal cortices. Although the outcomes were not compared between the DI and control groups, they were associated with improvements in general cognition and memory, which was interpreted as DI-induced compensatory enhancement (Qi et al., 2019).

The existing body of literature reveals inconsistencies that may be due to underlying mediators of interindividual variability, particularly in aged subjects (Angevaren et al., 2008). It is plausible to hypothesize that the effect of interventions is moderated by individual levels of cognitive reserve (CR) (Kim et al., 2021). Cognitive reserve refers to the facilitation of flexibility and the efficiency of neural networks to compensate for aging and increasing brain pathology (Stern et al., 2018). It may also be responsible for the optimization and recruitment of brain networks (Conti et al., 2021). In line with this notion, a study by Lin et al. (2021) recently demonstrated that, in patients with MCI, high CR (as proxied by years of education) can alleviate the impact of brain hypometabolism on executive function. This is partially achieved by increasing both rs-FC in brain regions involved in the DMN and resting-state dynamic functional connectivity (rs-dFC) in the right frontoparietal network and DMN. A dynamic functional connectivity (dFC) approach to resting-state connectivity accounts for the presence of temporal variability in rs-FC (Allen et al., 2014). Subsequently, it adds relevant information to the depiction of the dynamic nature of the brain (Calhoun et al., 2014). Thus, the evaluation of rs-dFC may improve our understanding of CR mechanisms in the context of interventions; more specifically, it may increase our understanding of how CR is implicated in DI-induced brain plasticity changes.

Cognitive reserve is usually proxied by demographic variables associated with lifetime enrichment. However, this becomes less accurate with aging, particularly in those with more depleted compensatory mechanisms. Therefore, we estimated a residual CR index as the unexplained variance of cognitive composite predicted from brain status (Reed et al., 2010) to evaluate individual variability in DI-induced effects. In this study, we hypothesized that the level of residual CR would moderate DI-induced changes in the dynamics of distinct rs-dFC states and that these changes would be associated with specific DI-induced cognitive outcomes.

## Methods

### Sample

A total of 99 community-dwelling, non-demented elderly (with MCI and healthy) subjects completed the study. All the subjects were over 60 years of age, without any medical, neurological, psychiatric, metabolic, or infectious disorders with an impact on cognition, such as major depression, drug and/or alcohol abuse, a history of traumatic brain injury, or any condition that would contraindicate DIs or MRI scanning. Subjects were randomized into a DI (*N* = 49) or a control (LAU) group (*N* = 50); see the study by Kropáčová et al. (2019) for the detailed enrollment and randomization processes. Then, each subject underwent a neuropsychological evaluation, MRI, and a physical fitness examination prior to the program and 6 months after the program completion. Demographic data included age, sex, education, clinical data (see section “Baseline Group Differences” and Table 1), and lifetime physical activities (Supplementary Table 3). Each subject signed the informed consent form in accordance with ethics codes and relevant regulations, and the study was then approved by the ethics committee of Masaryk University.

**TABLE 1:** Mean differences in demographic, cognitive, and dynamic functional connectivity (dFC) outcomes between the dance intervention (DI) and life-as-usual (LAU) groups at the baseline level.

			*n*	Mean	SD	*t*	*P*
	Age	LAU	32	69.027	6.072	–0.149	n.s.
		DI	36	69.236	5.467		
	Education	LAU	32	15.00	2.995	0.300	n.s.
		DI	36	14.81	2.390		
	CR	LAU	29	–1.086	74.000	–0.096	n.s.
		DI	33	0.955	91.645		
	FA	LAU	32	0.430	0.019	–0.406	n.s
		DI	36	0.432	0.017		
	MoCA	LAU	32	25.81	2.945	–2.282	0.026
		DI	36	27.39	2.749		
FCS1	DT	LAU	32	5.026	3.235	–0.037	n.s.
		DI	36	5.059	4.101		
	C	LAU	32	0.228	0.084	0.027	n.s.
		DI	36	0.228	0.088		
FCS2	DT	LAU	32	4.621	2.859	–0.957	n.s.
		DI	36	5.405	3.769		
	C	LAU	32	0.278	0.117	0.647	n.s.
		DI	36	0.259	0.118		
FCS3	DT	LAU	32	4.742	3.516	0.770	n.s.
		DI	36	4.181	2.444		
	C	LAU	32	0.171	0.064	–1.710	n.s.
		DI	36	0.199	0.071		
FCS4	DT	LAU	32	4.992	2.809	0.572	n.s.
		DI	36	4.584	3.043		
	C	LAU	32	0.193	0.101	0.936	n.s.
		DI	36	0.172	0.083		
FCS5	DT	LAU	32	3.812	3.812	0.393	n.s.
		DI	36	3.228	3.228		
	C	LAU	32	0.130	0.118	–0.422	n.s.
		DI	36	0.142	0.113		

*CR, residual index × 100; FA, mean fractional anisotropy of the white matter skeleton; education in years; DT, dwell time; C, coverage; MoCA, Montreal cognitive assessment test; DI, dance intervention group; LAU, life-as-usual group; n.s., non-significant; FCS, five dynamic resting brain states.*

### Dance Intervention

The intervention program was designed and supervised by experts at the Faculty of Sports Studies at Masaryk University. The intervention took 6 months for every 20 subjects and included three training units of 60 min per week. The duration of the study was 4 years. We aimed for a medium physical load intensity for each session, which included folk, country, African, Greek, and tango, each taught separately and developed over time into a final choreography. Only subjects who completed at least 60% of the DI program were included in the final cohort, resulting in an average compliance of 78.1%.

### Neuropsychological Examination

Global cognition (MoCA), activities of daily living, and five cognitive domains, i.e., memory, attention, executive, visuospatial, and language, were evaluated with complex neuropsychological testing (Supplementary Table 1). Based on our previous results from the final cohort (Kropáčová et al., 2019), we focused on tests of the executive domain [Tower of Hanoi (Humes et al., 1997), Five-Point Test (Tucha et al., 2012)], the attention domain [Symbol Search and Digit Span (Wechsler, 1997)], and on global cognition (Nasreddine et al., 2005).

### Physical Fitness Examination

Subjects were tested for physical parameters before and after the intervention (Šejnoha Minsterová et al., 2020). We evaluated body mass index, the 8-Foot Up-and-Go test, 30-Second Chair Stand test, 6-min Walk tests, and static (narrow and wide stance) and dynamic posture (standing up). The scores are compared in the Supplementary Table 2.

### fMRI Examination and Preprocessing

The subjects were scanned using the 3T Siemens Prisma MRI scanner (Siemens Corp., Erlangen, Germany) by employing various sequences, including T1 structural data for vertex-based and volumetric analyses. Scanning was also done through diffusion tensor imaging for tract-based spatial statistics (Kropáčová et al., 2019; Šejnoha Minsterová et al., 2020). For the current study, we used r-s fMRI data by employing gradient-echo echo-planar imaging sequences (200 scans, 34 transversal slices, slice thickness = 3.5 mm, TR = 1,990 ms, TE = 35 ms, FA = 70°, FOV = 192 mm, matrix size 64 × 64). Resting-state fMRI data were preprocessed using the SPM 12 toolbox (Wellcome Department of Imaging Neuroscience, UCL, UK) and Matlab 2014b (The MathWorks, Inc, Natick, United States). Preprocessing included realignment and unwarping, normalization into standard anatomical space (MNI), and spatial smoothing with 5 mm full width at half maximum (FWHM). More details on dealing with motion artifacts can be found in the Supplementary Material (page 1).

### Independent Component Analysis

The data from the subjects who had fMRI data that were of sufficient quality in both sessions were entered into a single group spatial independent component analysis (gsICA) (Calhoun et al., 2001) implemented in the Matlab-based toolbox, group ICA of fMRI Toolbox (GIFT) (TReNDS, GA, United States). The Infomax algorithm and ICASSO framework (Himberg et al., 2004) were then used to derive the maximally spatially group-specific independent components, while the group ICA (GICA) algorithm was used to render dataset-specific spatial maps and time-series. More details on ICA preprocessing can be found in the Supplementary Material (page 1).

### Dynamic Functional Network Connectivity

All 20 components were identified using the ICA. We visually inspected the spatial maps of the components and selected those representing functional networks. The sliding window correlation, i.e., Pearson’s approach (Chang et al., 2013; Shakil et al., 2016), between the temporal-series of nine selected independent components (ICs) were calculated. For each subject and condition, a window length of 60 s and 90% overlap formed a series of 56 correlation matrices 9 × 9.

Furthermore, *k*-means clustering was used to find recurring functional network states (Allen et al., 2018). Network state vectors, composed as the assignment of the correlation matrix of each subject in a time series to the nearest cluster by the *k*-means algorithm, were used to extract the parameters of network state dynamics (Díez-Cirarda et al., 2018). Parameters include (1) dwell time or mean duration, i.e., mean time of stay in a specific state and (2) time coverage, i.e., the percentage of data coverage by a specific state. For more details on the correlation matrices and clustering algorithm, see Supplementary Material (page 1).

### Residual CR

To obtain the residual CR proxy that reflects the current levels of pathological burden and cognitive performance, we calculated the residual difference between the observed memory performance and the prediction by (1) the hippocampal-to-intracranial volume ratio (Kropáčová et al., 2019), (2) fractional anisotropy (FA) of the white matter skeleton (Šejnoha Minsterová et al., 2020), and (3) age as a major risk factor for cognitive decline. Memory performance was selected to represent one of the most common domains to decline in healthy or pathological aging. The composite was calculated as the *z*-score of the Taylor figure (Warrington and Taylor, 1973) and Logical memory (Wechsler, 1997) (both immediate and delayed recall; see Supplementary Table 1). Intracranial volume as a denominator controlled for head size. The residual CR index was a continuous variable in which higher values indicate more reserve, i.e., capacity to compensate.

### Statistical Analyses

Between-group differences at baseline in dwell time and coverage of dFC states and demographics were compared using *t*-tests for continuous variables, chi-square tests for categorical variables, and Mann–Whitney *U* tests for mean ranks.

Linear mixed models (LMMs) were computed to assess the interaction effect between time^∗^group^∗^CR on the dwell time and coverage of the rs-dFC states. These LMMs included (1) random intercepts to account for variability across individuals, (2) time as a repeated measure with an unstructured covariance type to account for high correlations between outcome variables measured at two time points, and (3) no random factors, as they did not improve the fit of models. The first-level, basic LMM included only fixed factors with the main effects of time (dichotomous), cognitive reserve (continuous variable), and group (dichotomous, DI vs. LAU). Age and sex were selected as covariates and included as main effects. The second-level LMMs were run with the same main effects and interactions of time^∗^group and time^∗^group^∗^CR. The fit of the models with significant interaction effects was compared with the basic model using the X^2^-change of maximum likelihood estimation (–2 LL).

To probe significant three-way interactions from the LMMs, moderation analyses were computed to test the effect of CR (moderator variable) on the relationship between the program and the change in dwell time and/or coverage of the rs-dFC states. Using the Johnson–Neyman output made modeling the effects of different levels of the CR moderator possible, consequently simplifying the interpretation of interactions based on zones of significance without necessitating multiple tests of single main effects. The HC3 option (Davidson-MacKinnon) was also selected for its heteroscedasticity-consistent standard errors, while only the continuous variables that predicted the outcome were centered.

Finally, we conducted exploratory two-tailed partial correlations for the purposes of interpretation by accounting for CR between changes in the rs-dFC parameters and cognitive tests of interest in the DI group. The rs-dFC states that significantly changed as a result of the time^∗^group^∗^CR interaction were the only one that were correlated, as we were specifically interested in the unique relationship between the rs-dFC states and cognitive tests. Furthermore, changes in variables of interest for moderation and correlations were computed as timepoint_2_ (a follow-up) − timepoint_1_ (baseline).

## Results

### Subjects

From the original cohort of 120 subjects, we excluded 21 who did not complete at least 60% of the DI. More subjects were later excluded due to insufficient fMRI data quality. The final dataset was comprised of 36 DI and 32 LAU subjects; a total of 68 × 2 = 136 datasets were entered into further analyses. For the demographic, clinical, and MRI data, see Table 1.

### Independent Component Analysis

**FIGURE 1:**
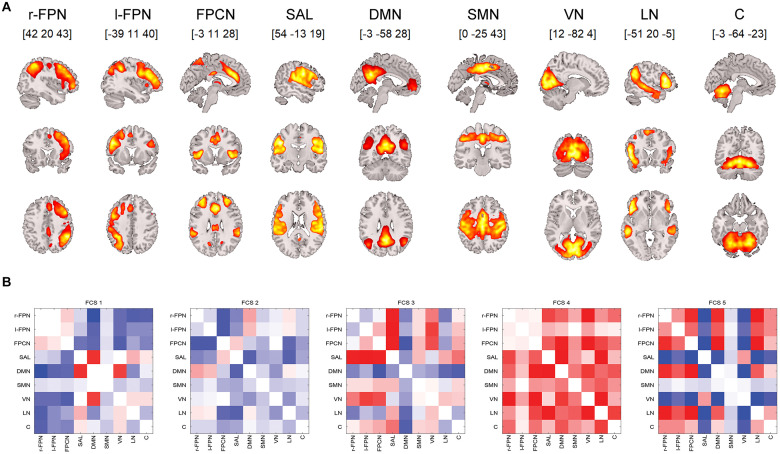
**(A)** Nine ICA components analyzed for the resting-state dynamic functional connectivity. **(B)** Five identified rs-dFC states (1–5 from the left). Each matrix depicts mutual correlations between each component identified using the ICA. Dark blue suggests a high negative correlation, and dark red suggests a high positive correlation. Note: rs-dFC, resting state dynamic functional connectivity states; r/l-FPN, right/left frontoparietal network; FPCN, frontoparietal control network; SAL, salience (insulo-opercular) network; DMN, default-mode network; SMN, sensorimotor network; VN, visual network; LN, language network; C, cerebellum.

In this study, we selected nine components that represented functional networks: the cerebellum, DMN, visual network (VN), right and left frontoparietal network (r/l-FPN), language network (LN), salience (insulo-opercular) network (SAL), frontoparietal control network (FPCN), and sensorimotor network (SMN) (Figure 1A).

### Dynamic Functional Connectivity States

Altogether, five rs-dFC states (FCS1–5) were identified (Figure 1B). For the baseline differences in coverage and dwell time in the DI and LAU groups, see Table 1.

The first functional connectivity state (FCS1) was characterized by sparsely connected networks, where the DMN significantly correlated with both salience and visual networks, thus suggesting bottom-up processing and readiness to salient stimuli. A sparsely connected FCS2 state was characterized by overall negative correlations, particularly between the DMN and SAL, suggesting a disconnected resting state. The FCS3 state was characterized by anticorrelations between task-positive and task-negative networks and resembled a typical “mind wandering” resting state. In contrast to the three FCSs, the last two identified states were highly interconnected with FCS4, suggesting both top-down and bottom-up processing readiness. Additionally, FCS5 was partially inverse to FCS1 but displayed disconnection between the DMN and SAL, thus resembling a resting state.

### Baseline Group Differences

The two experimental groups were equivalent in terms of age, education, residual CR, and parameters of the identified FCSs at the baseline (see Table 1). Similarly, the proportion of subjects with MCI (*n* = 21) [*X*^2^(1) = 1.24; *p* = 0.27] and male-to-female ratio [15:51; *X*^2^(1) = 3.02; *p* = 0.082] did not significantly differ between the DI and the LAU groups. The baseline comparisons of the tests of interest from the attention and executive domains between DI and LAU also did not differ (*p* > 0.05, data not shown). There were no between-group differences in baseline fitness parameters (Supplementary Table 2) or in lifetime engagement in aerobic (*U* = 540, *Z* = −0.23, *p* = 0.82) and anaerobic activities (*U* = 471, Z = −1.22, *p* = 0.22) (details reported in Supplementary Table 3).

### Analyses of rs-dFC States

There were no significant interactions in the FCS2, FCS3 and FCS5 predictions. For a detailed report of the LMM fixed effects in all five states, see Supplementary Table 4.

#### FCS1 State

**FIGURE 2:**
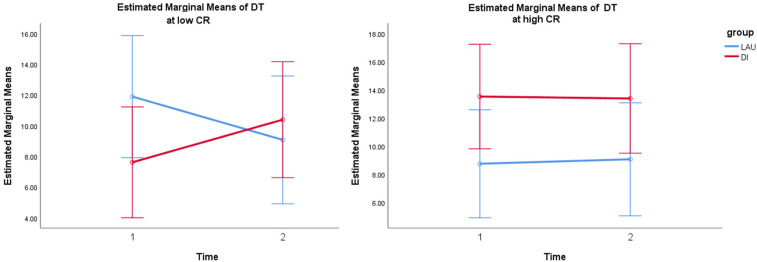
Simple slope analysis of time*group interaction in low vs. high CR (based on median-split grouping) depicts significant interaction at lower values of CR. Error bars: 95% CI. Note: DT, dwell time; CR, cognitive reserve; LAU, life-as-usual; DI, dance intervention.

The dwell time of the FCS1 state was significantly predicted by a model with the time^∗^group^∗^CR interaction term, with a significantly better fit [*X*^2^(3) = 11.46, *p* < 0.01] than the basic model with only the main effects and covariates –2 LL(9) = 675.908, and also in comparison to a model with the same main effects and the simple time^∗^group interaction term [*X*^2^(2) = 10.56, *p* < 0.01]. Only the interaction between time^∗^group^∗^CR significantly predicted the change in the dwell time of FCS1 *F*(3,73) = 4.17, *p* = 0.009 (see Supplementary Table 4), but not the main effects of predictors and covariates. The follow-up Johnson–Neyman technique to determine zones of significance revealed that DIs positively predicted FCS1 dwell time increases at lower values of residual CR [from approximately –0.5 SD (*t* = 2.01, *p* = 0.05) to the lowest CR levels (*t* = 2.31, *p* = 0.024)] (Figure 2).

Similarly, the coverage of FCS1 was significantly predicted by a model with the time^∗^group^∗^CR interaction, which was a significantly better fit [*X*^2^(3) = 8.72, *p* < 0.05] than both the basic model –2 LL(9) = –240.386 and a model with the simple time^∗^group interaction term [*X*^2^(2) = 8.7, *p* < 0.05]. In this model, only the interaction between time^∗^group^∗^CR significantly predicted the coverage of FCS1 *F*(3,70) = 3.13, *p* = 0.031 (see Supplementary Table 4), whereas the main effects of the predictors and covariates were not significant. The follow-up Johnson–Neyman technique to determine zones of significance revealed that DI had a positive significant effect on the increased coverage of FCS1 at lower values of residual CR [from approximately –1 SD (*t* = 2.01, *p* = 0.05) to the lowest CR levels (*t* = 2.32, *p* = 0.024)].

In other words, both the dwell time and coverage change of the FCS1 state were moderated by CR and increased after the DI in subjects with low residual CR.

According to the partial correlation results in the DI group, the increase in the dwell time of FCS1 was associated with improvement in the Symbol Search task (*r* = 0.41, *p* = 0.023).

#### FCS4 State

The linear mixed models of the dwell time of FCS4 did not reveal any significant predictors of change. However, the coverage was significantly predicted by a model with the time^∗^group^∗^CR interaction, which was a significantly better fit [*X*^2^(2) = 7.06, *p* < 0.05] than a basic model with only the main effects of fixed factors and covariates –2 LL(9) = –256.221, but not in comparison with a model with the same main effects and simple time^∗^group interaction term [*X*^2^(1) = 3.57, *p* = 0.059] (Supplementary Table 4). The time^∗^group^∗^CR interaction term had the greatest effect *F*(3,72) = 2.61, *p* = 0.058. When proceeding with a simpler moderation analysis, Johnson–Neyman zones of significance supported the abovementioned interaction effect [*t*(58) = 2.11, *p* = 0.039]. Furthermore, DIs also positively predicted FCS4 coverage changes from moderate (*t* = 2.01, *p* = 0.05) to the highest levels of residual CR (*t* = 2.58, *p* = 0.012).

In other words, the coverage change of the FCS4 state was moderated by CR and increased after the DI in subjects with moderate to the highest residual CR levels.

Partial correlations in the DI group did not yield any significant results for the association between FCS4 and cognitive changes.

## Discussion

The novelty of our study stems from the evaluation of the temporal dynamics of inter-network connectivity between several large-scale brain networks at the baseline and following a 6-month DI in non-demented (HC and MCI) elderly as compared with a control condition (LAU). Cognitive reserve is involved in the optimal recruitment of brain networks, which is important to maintain cognitive performance and compensate for cognitive decline. By implementing the individual residual levels of CR that account for brain pathology and memory performance, we were able to evaluate how current CR capacity can moderate the effects of a comprehensive DI program on the dwell time and coverage of several brain rs-dFC states.

This study reported two main findings. The first finding was related to the temporal dynamics of the sparsely connected rs-dFC state 1, which was characterized by a significant correlation between the SAL and the DMN. The DMN in turn positively correlated with the VN, whereas the FPCN was significantly anticorrelated with both the SAL and DMN and the cerebellar, VN, and language networks. The dwell time and coverage of this state increased in the DI group as compared with the LAU group, but only in people with low residual CR, invariantly of age and sex. Increased engagement of the insula and decreased involvement of the FPCN in individuals with low CR were previously shown in a study by Huang et al. (2019) on depressed elderly subjects during an emotional interference task. It has been proposed that the insula, as part of the SAL, is involved in detecting and filtering salient stimuli, subsequently guiding behavior by engaging in the dynamic coordination of large-scale networks (Seeley et al., 2007; Uddin, 2015), and switching between task-negative (DMN) and task-positive (FPN) networks (Menon and Uddin, 2010). Other task-based fMRI studies have shown the role of the ACC and the insula in experiencing thirst, hunger, pain, embarrassment, and other emotions (Craig, 2002; Critchley, 2005). Therefore, the results of this study suggested that, by prolonging the coverage and dwell time of this particular dynamic state, the DI enhanced readiness to the bottom-up processing of new relevant stimuli in low-CR individuals by drawing attention to input signals from the internal environment (SAL and DMN) and the external environment (VN).

The increase in dwell time of the rs-dFC state was accompanied by improvements in the Symbol Search score, an established measure of psychomotor and visuomotor processing speed, visual discrimination, and attention. This is clinically relevant because the test has a strong negative relationship with age due to declining processing speed (Joy and Fein, 2001). The finding that Symbol Search performance was more readily improved with bottom-up processing accords with the notion that low-CR individuals rely on less effective bottom-up information processing, probably due to their already depleted top-down processing (Steffener and Stern, 2012). Interestingly, the dwell time and coverage of state 1 in high-CR subjects remained stable in time in both the DI and LAU groups.

The second finding indicated that subjects with high residual CR displayed DI-induced increases in the coverage of the highly interconnected state 4, in which the SAL was positively correlated with the DMN and the DMN also positively correlated with the FPCN. Thus, FCS4 may represent increased readiness for top-down information processing in high-CR individuals, which was enhanced by the DI. Other authors have supported the notion that the cognitive functioning in high-CR individuals employs more prefrontal and frontoparietal networks, e.g., in Alzheimer’s disease (Colangeli et al., 2016), and engages in attentional, executive (Decety and Lamm, 2006), and complex cognitive operations (Song et al., 2017). In the FCS1, the DMN was also correlated with the VN, although less strongly than with the FPCN, which suggested readiness for bottom-up processing in addition to top-down cognitive control. However, the changes in the dynamic state 4 in high-CR individuals did not correlate with changes in cognitive tests. Such a result may be a consequence of the already optimized cognitive functioning among high-CR subjects and/or of the lower sensitivity of the administered tests.

Cognitive reserve is defined as an active process in which flexible and efficient neural networks compensate for age- or disease-related neurocognitive declines (Stern et al., 2018). The current results shed light on how CR moderates intervention-induced rs-dFC changes that underpin optimized cognitive processing in non-demented elderly subjects. Specifically, we observed that the DI-induced benefits in cognitive processing were dependent on the CR level and linked to distinct and differential neural mechanisms as assessed by rs-dFC. In low-CR subjects, the DI enhanced bottom-up processing, while in high-CR individuals, the DI particularly affected top-down processing. These findings could aid in individualization and evaluation in future intervention studies, as they underscore the importance of monitoring the individual capacity for brain plasticity changes and network optimization due to interventions.

This current study has its limitations, which the authors recognize. First, we used a non-active “life-as-usual” control group; thus, we were unable to control for other significant factors such as the effect of socialization. Second, the subjects with MCI could not be analyzed separately due to low representation. Future studies may also confirm our findings in patients with MCI and dementia.

Dynamic resting-state functional connectivity offers a new approach to studying functional connectivity on time-varying frameworks of coupling among brain networks. Therefore, it allows for the representation of multiple brain states (Allen et al., 2014). Previous studies utilizing this approach linked CR in young professional chess players to enhanced global dynamic fluidity and a higher number of occupied states (Premi et al., 2020). Thus, this study employed rs-FC and brain state dynamics to study controlled pre-to-post intervention effects in the context of age-related CR depletion.

The results of this study clearly supported our hypothesis that individual differences in CR moderate the brain plasticity outcomes of an intervention program. Specifically, it was found that the changes in rs-dFC observed in the DI group subjects were CR-specific and behaviorally relevant. They reflected enhanced bottom-up cognitive processing in the low-CR subjects, which was probably due to the depleted capacity of the frontoparietal networks, and enhanced top-down information processing in those with high residual CR. The study provided the first pieces of evidence for individual variability in DI-induced rs-dFC changes in aging brains and offered new insight into the role of DIs in enhancing brain function in aged non-demented subjects.

## Data Availability Statement

The raw data supporting the conclusions of this article will be made available by the authors, upon reasonable request.

## Ethics Statement

The studies involving human participants were reviewed and approved by Masaryk University. The patients/participants provided their written informed consent to participate in this study.

## Author Contributions

KM, IR, MP, PŠ, PV, AS, and RG: study conception and design. KM, MP, PŠ, PV, AS, and RG: data collection. KM, ML, RM, and IR: analysis and interpretation of results. KM: draft manuscript preparation. All authors reviewed the results and approved the final version of the manuscript.

## Conflict of Interest

The authors declare that the research was conducted in the absence of any commercial or financial relationships that could be construed as a potential conflict of interest.

## Publisher’s Note

All claims expressed in this article are solely those of the authors and do not necessarily represent those of their affiliated organizations, or those of the publisher, the editors and the reviewers. Any product that may be evaluated in this article, or claim that may be made by its manufacturer, is not guaranteed or endorsed by the publisher.
