# Supplementary material for: Dynamic Functional Connectivity Signifies the Joint Impact of Dance Intervention and Cognitive Reserve
Source: Front Aging Neurosci. 2021 Sep 10;13:724094. doi: 10.3389/fnagi.2021.724094 (PMC8462054; doi:10.3389/fnagi.2021.724094)
Supplement: Supplementary file 1 [file Data_Sheet_1.docx]

Supplementary Material

## Preprocessing of fMRI data

We checked a level of motion in fMRI data and those subjects who did not meet pre-set criteria were removed from further analyses. The criteria were: a) frame –wise displacement (FD) throughout all dataset bellow 3mm; b) more than 2.5% of scans showed FD > 0.75mm (Power et al., 2012). The datasets that passed were corrected by scrubbing the scans with FD > 0.75mm (Power et al., 2012). Finally, we applied a linear regression to filter out physiological noise using the six movement parameters (from realignment step), FD and signals drawn from cerebrospinal fluid and white matter.

## Independent component analysis for functional connectivity changes

The details on group spatial ICA: 1) preprocessing: removing mean from each voxel’s timeseries; 2) data reduction: two successive Principal Component Analysis (PCA), the first PCA reduced each dataset into a number of components set according to the maximal Minimum Description Length (MDL) estimate over all datasets (Li et al., 2007); the resulting components were reduced by the second PCA with number of components set to the median over MDL estimates; 3) ICA estimation: the Infomax algorithm were run 20 times with random initialization under ICASSO framework; those components were considered as reliable and used in further analyses that were stable over Infomax runs (minimal/maximal cluster size of 16/24); 4) back reconstruction: we employed GICA algorithm

## Dynamic Functional Network Connectivity

After the correlation matrices were formed, the mean correlation matrix was subtracted from each matrix. A series of de-meaned matrices from each subject and condition were concatenated across a third (temporal) dimension.

K-means clustering applied on the concatenated matrices was used to find recurring functional network states (Allen et al., 2018). The optimal number of clusters was determined by the mean criterion, which contained measures of the Calinski-Harabasz index, the Davies-Bouldin index, and silhouette values. The clustering algorithm was repeated 1000× with random initialization of centroid positions. The final cluster centroids represent functional network states.

# Supplementary Tables

**TABLE S1|** Neuropsychological assessment. Tests and cognitive domains.

| **Domains** | **Test/subtest** |
| --- | --- |
| Global cognition | Montreal Cognitive Assessment (Nasreddine et al., 2005) |
| Memory | Taylor figure test/ recall 3 min after copy (Warrington, Taylor, 1973) |
|  | Taylor figure test/ recall 30 min after copy (Warrington, Taylor, 1973) |
|  | WMS III/ Logical memory immediate recall (Wechsler, 1997) |
|  | WMS III/ Logical memory recall after 30 min (Wechsler, 1997) |
| Attention | WAIS III/ Symbol search (Wechsler, 1997) |
|  | WAIS III/ Digit span (Wechsler, 1997) |
| Executive function | Tower of Hanoi/ 3 discs (Humes et al., 1997) |
|  | Tower of Hanoi/ 4 discs (Humes et al., 1997) |
|  | Five-Point Test (Tucha et al., 2012) |
| Visuospatial function | Judgement of Line Orientation test (Benton et al., 1994) |
|  | Taylor figure/ copy (Warrington, Taylor, 1973) |
| Self-dependence | Bristol Activities of Daily Living Scale (Bucks et al., 1996) |
| Depression | Beck Depression Inventory II (Beck et al., 1996) |

**TABLE S2|** Between-group comparison of physical fitness at baseline.

|  | | | **n** | **Mean** | **SD** | **t** | **p** |
| --- | --- | --- | --- | --- | --- | --- | --- |
|  | **BMI** | LAU | 32 | 26.459 | 3.772 | -0.352 | n.s. |
|  |  | DI | 35 | 26.875 | 5.619 |  |  |
|  | **6-minute walk** | LAU | 32 | 561.875 | 92.403 | -0.133 | n.s. |
|  |  | DI | 35 | 564.714 | 82.095 |  |  |
|  | **30 Stand Chair test** | LAU | 32 | 17.094 | 5.232 | 1.607 | n.s. |
|  |  | DI | 35 | 15.400 | 3.247 |  |  |
|  | **8 Foot Up and Go task** | LAU | 32 | 5.266 | 1.448 | 0.001 | n.s. |
|  |  | DI | 35 | 5.266 | 1.286 |  |  |
| **Static posture** | **Wide stance** | LAU | 32 | 2.681 | 2.604 | 0.929 | n.s. |
|  |  | DI | 35 | 2.212 | 1.396 |  |  |
|  | **Wide stance** | LAU | 32 | 2.136 | 0.672 | 0.137 | n.s. |
|  |  | DI | 35 | 2.108 | 0.955 |  |  |
|  | **Wide stance** | LAU | 32 | 1.462 | 0.907 | 0.772 | n.s. |
|  |  | DI | 35 | 1.316 | 0.626 |  |  |
|  | **Narrow stance** | LAU | 32 | 6.814 | 5.532 | 1.172 | n.s. |
|  |  | DI | 35 | 5.463 | 3.818 |  |  |
|  | **Narrow stance** | LAU | 32 | 2.726 | 1.298 | 0.551 | n.s. |
|  |  | DI | 35 | 2.568 | 1.049 |  |  |
|  | **Narrow stance** | LAU | 32 | 2.829 | 1.045 | 1.261 | n.s. |
|  |  | DI | 35 | 2.527 | 0.914 |  |  |
|  | **Wide stance closed eyes** | LAU | 32 | 3.302 | 3.630 | 1.436 | n.s. |
|  |  | DI | 35 | 2.312 | 1.780 |  |  |
|  | **Wide stance closed eyes** | LAU | 32 | 2.492 | 1.023 | 1.198 | n.s. |
|  |  | DI | 35 | 2.197 | 0.986 |  |  |
|  | **Wide stance closed eyes** | LAU | 32 | 1.480 | 0.882 | 1.398 | n.s. |
|  |  | DI | 35 | 1.223 | 0.572 |  |  |
|  | **Narrow stance closed eyes** | LAU | 32 | 10.176 | 9.326 | 0.799 | n.s. |
|  |  | DI | 35 | 8.666 | 5.909 |  |  |
|  | **Narrow stance closed eyes** | LAU | 32 | 3.359 | 1.654 | 0.746 | n.s. |
|  |  | DI | 35 | 3.100 | 1.162 |  |  |
|  | **Narrow stance closed eyes** | LAU | 32 | 3.404 | 1.394 | -0.065 | n.s. |
|  |  | DI | 35 | 3.425 | 1.314 |  |  |
| **Dynamic posture** | **Stand-up** | LAU | 32 | 43.689 | 38.843 | 0.639 | n.s. |
|  |  | DI | 35 | 37.041 | 45.680 |  |  |
|  | **Stand-up** | LAU | 32 | 8.140 | 3.755 | 1.135 | n.s. |
|  |  | DI | 35 | 7.148 | 3.400 |  |  |
|  | **Stand-up** | LAU | 32 | 5.824 | 3.877 | 0.575 | n.s. |
|  |  | DI | 35 | 5.274 | 3.940 |  |  |

*BMI: body mass index; DI: dance intervention group; LAU: life-as-usual group; n.s.: non-significant*

**TABLE S3|** Between-group comparison of lifetime engagement in physical activities assessed before the intervention.

| **Activity** | **Type** | **N** | | **Median number of activities** | **Mean Rank** | **Sum of ranks** |
| --- | --- | --- | --- | --- | --- | --- |
| Racket sports | **Aerobic activities** | DI | 36 | 2 | 33.50 | 1206.0 |
| Ball games |  | LAU | 31 | 2 | 34.58 | 1072.0 |
| Water sports |  | Total | 67 | 2 |  |  |
| Winter sports |  |  |  |  |  |  |
| Jogging |  |  |  |  |  |  |
| Cycling |  |  |  |  |  |  |
| Hiking |  |  |  |  |  |  |
| Cardio |  |  |  |  |  |  |
| Athletics | **Anaerobic activities** | DI | 36 | 1 | 36.42 | 1311.0 |
| Fitness |  | LAU | 31 | 1 | 31.19 | 967.0 |
| Combative sports |  | Total | 67 | 1 |  |  |

*DI: dance intervention group; LAU: life-as-usual group; n.s.: non-significant*

**TABLE S4|** Tests of fixed effects for dynamic brain states as dependent variables.

| **Model terms** | **Numerator df** | **Denominator df** | **F** | ***p*** |
| --- | --- | --- | --- | --- |
| **FCS1 dwell time** | | | | |
| Intercept | 1 | 61.85 | 2.91 | .093 |
| Time | 1 | 62.22 | .01 | .905 |
| Group | 1 | 62.55 | 2.14 | .149 |
| CR | 1 | 63.51 | .02 | .879 |
| Sex | 1 | 62.18 | 2.53 | .117 |
| Age | 1 | 61.86 | .05 | .829 |
| Time*group*CR | 3 | 72.73 | 4.17 | .009 |
| **FCS1 coverage** | | | | |
| Intercept | 1 | 61.91 | 2.53 | .117 |
| Time | 1 | 62.14 | .41 | .527 |
| Group | 1 | 62.58 | .07 | .789 |
| CR | 1 | 63.28 | .54 | .465 |
| Sex | 1 | 62.21 | 1.04 | .312 |
| Age | 1 | 61.90 | .88 | .352 |
| Time*group*CR | 3 | 70.42 | 70.13 | .031 |
| **FCS4 coverage** | | | | |
| Intercept | 1 | 60.33 | 4.88 | .031 |
| Time | 1 | 60.40 | 3.32 | .077 |
| Group | 1 | 60.98 | .01 | .957 |
| CR | 1 | 62.00 | .01 | .967 |
| Sex | 1 | 60.63 | .19 | .664 |
| Age | 1 | 60.35 | .74 | .393 |
| Time*group*CR | 3 | 71.53 | 2.61 | .058 |
| **FCS2 dwell time** | | | | |
| Intercept | 1 | 61.16 | 2.12 | .151 |
| Time | 1 | 61.70 | 5.59 | .021 |
| Group | 1 | 61.52 | .56 | .457 |
| CR | 1 | 61.88 | 2.21 | .142 |
| Sex | 1 | 61.21 | 2.22 | .142 |
| Age | 1 | 61.10 | .02 | .882 |
| **FCS2 coverage** | | | | |
| Intercept | 1 | 61.16 | .54 | .464 |
| Time | 1 | 61.15 | 4.46 | .039 |
| Group | 1 | 61.65 | .96 | .331 |
| CR | 1 | 62.03 | 3.44 | .068 |
| Sex | 1 | 61.31 | 2.54 | .116 |
| Age | 1 | 61.19 | .62 | .436 |
| **FCS3 dwell time** | | | | |
| Intercept | 1 | 58.09 | 2.37 | .129 |
| Time | 1 | 58.46 | .08 | .784 |
| Group | 1 | 58.62 | 5.27 | .025 |
| CR | 1 | 59.04 | .36 | .550 |
| Sex | 1 | 58.26 | .25 | .618 |
| Age | 1 | 59.13 | .02 | .892 |
| **FCS3 coverage** | | | | |
| Intercept | 1 | 61.79 | .98 | .326 |
| Time | 1 | 61.47 | .66 | .421 |
| Group | 1 | 62.09 | .06 | .806 |
| CR | 1 | 62.42 | .26 | .612 |
| Sex | 1 | 61.85 | .03 | .858 |
| Age | 1 | 61.75 | 1.01 | .318 |
| Time*group | 1 | 61.47 | 5.06 | .028 |
| **FCS5 dwell time** | | | | |
| Intercept | 1 | 62.10 | 10.26 | .002 |
| Time | 1 | 62.83 | 3.11 | .083 |
| Group | 1 | 62.48 | .09 | .767 |
| CR | 1 | 62.81 | 3.43 | .069 |
| Sex | 1 | 62.19 | 2.14 | .149 |
| Age | 1 | 62.09 | 4.14 | .046 |
| **FCS5 coverage** | | | | |
| Intercept | 1 | 59.58 | 5.40 | .024 |
| Time | 1 | 59.38 | 6.22 | .015 |
| Group | 1 | 60.01 | .47 | .497 |
| CR | 1 | 60.36 | 1.24 | .270 |
| Sex | 1 | 59.72 | 1.40 | .242 |
| Age | 1 | 59.61 | 1.40 | .241 |

*Note: Linear model of predictors of dynamic resting-state functional connectivity changes in dwell time and coverage of FCS 1-5. Results are fixed effects from all significant linear mixed models estimating the effect of CR, group (DI, LAU), time (two time points), and the covariates of sex and age on the FCS 1-5. Note that the three-way interaction effect was significant for only the dwell duration of FCS1, and coverage of FCS1. CR, cognitive reserve; FCS, dynamic resting-state functional connectivity brain states.*
